# Aqua­(pyridine-3-carb­oxy­lic acid-κ*N*)(pyridine-2,6-dicarboxyl­ato-κ^3^ *O* ^2^,*N*,*O* ^6^)copper(II) monohydrate

**DOI:** 10.1107/S160053681101926X

**Published:** 2011-05-28

**Authors:** Ting Ting Chen, Yan Fang Shang, Xia Xi, Yue Hua Zhang, Nan Ping Wang

**Affiliations:** a School of Chemistry and Chemical Engineering, Nantong University, Nantong 226019, People’s Republic of China

## Abstract

In the title Cu^II^ complex, [Cu(C_7_H_3_NO_4_)(C_6_H_5_NO_2_)(H_2_O)]·H_2_O, the environment of the Cu^2+^ ion is a distorted square pyramid with the axial site occupied by the O atom from the coordinated water mol­ecule and the square base formed by two O and two N atoms from the tridentate anion and the neutral monodentate pyridine-3-carboxylic acid ligand. O—H⋯O hydrogen bonds, as well as π–π inter­actions [centroid–centroid distance = 3.945 (3) Å] contribute to the stabilization of this structure.

## Related literature

For the use of transition-metal–carboxyl­ate systems in supra­molecular chemistry and functional materials, see: MacDonald *et al.* (2000 ▶); Siddiqui *et al.* (2008 ▶); Custelcean & Gorbunova (2005 ▶). For a description of the geometry of complexes with five-coordinate metal atoms, see: Addison *et al.* (1984 ▶).
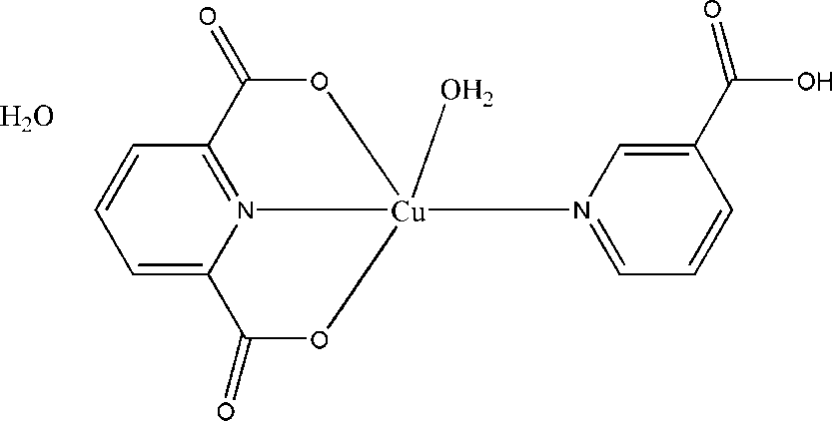


## Experimental

### 

#### Crystal data

[Cu(C_7_H_3_NO_4_)(C_6_H_5_NO_2_)(H_2_O)]·H_2_O
*M* *_r_* = 387.79
Triclinic, 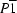

*a* = 7.3241 (12) Å
*b* = 9.5290 (16) Å
*c* = 11.1895 (18) Å
α = 107.471 (3)°
β = 92.822 (3)°
γ = 101.547 (3)°
*V* = 724.8 (2) Å^3^
*Z* = 2
Mo *K*α radiation
μ = 1.56 mm^−1^
*T* = 293 K
0.34 × 0.28 × 0.24 mm

#### Data collection

Bruker APEXII CCD diffractometer
Absorption correction: multi-scan (*SADABS*; Bruker, 2001 ▶) *T*_min_ = 0.612, *T*_max_ = 0.674
3704 measured reflections
2544 independent reflections
2036 reflections with *I* > 2σ(*I*)
*R*_int_ = 0.058

#### Refinement

*R*[*F* ^2^ > 2σ(*F* ^2^)] = 0.047
*wR*(*F* ^2^) = 0.089
*S* = 1.01
2544 reflections
218 parameters
H-atom parameters constrained
Δρ_max_ = 0.61 e Å^−3^
Δρ_min_ = −0.38 e Å^−3^

### 

Data collection: *APEX2* (Bruker, 2001 ▶); cell refinement: *SAINT* (Bruker, 2001 ▶); data reduction: *SAINT*; program(s) used to solve structure: *SHELXS97* (Sheldrick, 2008 ▶); program(s) used to refine structure: *SHELXL97* (Sheldrick, 2008 ▶); molecular graphics: *SHELXTL* (Sheldrick, 2008 ▶); software used to prepare material for publication: *SHELXTL* .

## Supplementary Material

Crystal structure: contains datablocks I, global. DOI: 10.1107/S160053681101926X/yk2006sup1.cif

Structure factors: contains datablocks I. DOI: 10.1107/S160053681101926X/yk2006Isup2.hkl

Additional supplementary materials:  crystallographic information; 3D view; checkCIF report

## Figures and Tables

**Table 1:** Hydrogen-bond geometry (Å, °)

*D*—H⋯*A*	*D*—H	H⋯*A*	*D*⋯*A*	*D*—H⋯*A*
O5—H5*B*⋯O1*W*^i^	0.85	1.87	2.715 (4)	175
O5—H5*A*⋯O7^i^	0.85	2.08	2.888 (4)	158
O6—H6⋯O4^ii^	0.82	1.79	2.586 (4)	164
O1*W*—H1*WC*⋯O3^iii^	0.85	2.14	2.983 (4)	172
O1*W*—H1*WD*⋯O2	0.85	1.95	2.775 (4)	163

Symmetry codes: (i) 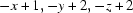; (ii) 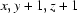; (iii) 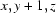.

**Fig. 1.**
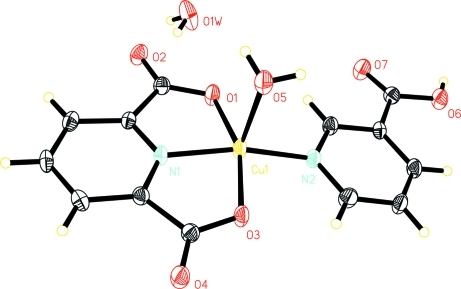
The molecular structure of the title complex, with atomic labels and 30% probability displacement ellipsoids for non-H atoms.

**Fig. 2.**
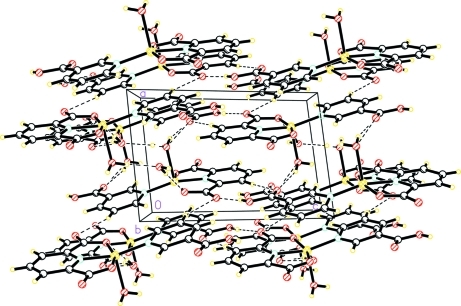
The three-dimensional packing diagram of title compound, showing O—H···O and C—H···O hydrogen-bonds (dashed lines) and π–π interactions.

## supplementary crystallographic information

### Comment

In recent years, the chemistry of transition metal carboxylate systems is of great interest because of their extensive usage in supramolecular chemistry and functional materials (Custelcean & Gorbunova, 2005; MacDonald, *et al.*, 2000; Siddiqui, *et al.*, 2008). Here, we report a new Cu^II^complex with pyridine-2,6-dicarboxylic acid and pyridine-3-carboxylic acid.

In this complex, the Cu^2+^ ion is coordinated by one oxygen atom from water molecule, two oxygen atoms from the dianion of pyridine-2,6-dicarboxylic acid, and two nitrogen atoms from pyridine-2,6-dicarboxylate and pyridine-3-carboxylic acid, respectively (Fig. 1). Thus, the coordination environment of Cu^2+^ ion is a distorted square pyramid, as indicated by τ value of 0.06 (Addison, *et al.*, 1984). The asymmetric units are linked into three-dimensional structure through C—H···O and O—H···O hydrogen bonds and π-π interactions between the neighbouring pyridine rings with centroid–centroid distance of 3.945 (3) Å (Fig. 2).

### Experimental

A mixture of Cu(CH_3_COO)_2_^.^H_2_O (0.5 mmol), pyridine-2,6-dicarboxylic acid (0.5 mmol), pyridine-3-carboxylic acid (0.5 mmol) in H_2_O (4 ml) and CH_3_OH (4 ml) was adjusted to pH *ca* 7.5 by triethylamine, sealed in a Teflon lined stainless steel container and heated at 140 °C for 3 days. After the sample was cooled to room temperature, blue crystals were collected with a yield of 30%.

### Refinement

All H atoms were refined using a riding model. C—H values were set to 0.93 Å with *U*_iso_(H) =1.2 *U*_eq_(C), and O—H values were set to 0.82 to 0.85 Å with *U*_iso_(H) =1.5 *U*_eq_(N).

### Crystal data

**Table:**

[Cu(C_7_H_3_NO_4_)(C_6_H_5_NO_2_)(H_2_O)]·H_2_O	*Z* = 2
*M**_r_* = 387.79	*F*(000) = 394
Triclinic, *P*1	*D*_x_ = 1.777 Mg m^−^^3^
Hall symbol: -P 1	Mo *K*α radiation, λ = 0.71073 Å
*a* = 7.3241 (12) Å	Cell parameters from 3246 reflections
*b* = 9.5290 (16) Å	θ = 1.9–28.0°
*c* = 11.1895 (18) Å	µ = 1.56 mm^−^^1^
α = 107.471 (3)°	*T* = 293 K
β = 92.822 (3)°	Block, blue
γ = 101.547 (3)°	0.34 × 0.28 × 0.24 mm
*V* = 724.8 (2) Å^3^	

### Data collection

**Table:**

Bruker APEXII CCD diffractometer	2544 independent reflections
Radiation source: fine-focus sealed tube	2036 reflections with *I* > 2σ(*I*)
graphite	*R*_int_ = 0.058
φ and ω scans	θ_max_ = 25.1°, θ_min_ = 1.9°
Absorption correction: multi-scan (*SADABS*; Bruker, 2001)	*h* = −8→8
*T*_min_ = 0.612, *T*_max_ = 0.674	*k* = −11→7
3704 measured reflections	*l* = −13→12

### Refinement

**Table:**

Refinement on *F*^2^	Primary atom site location: structure-invariant direct methods
Least-squares matrix: full	Secondary atom site location: difference Fourier map
*R*[*F*^2^ > 2σ(*F*^2^)] = 0.047	Hydrogen site location: inferred from neighbouring sites
*wR*(*F*^2^) = 0.089	H-atom parameters constrained
*S* = 1.01	*w* = 1/[σ^2^(*F*_o_^2^) + (0.0156*P*)^2^ + 0.0*P*] where *P* = (*F*_o_^2^ + 2*F*_c_^2^)/3
2544 reflections	(Δ/σ)_max_ = 0.001
218 parameters	Δρ_max_ = 0.61 e Å^−^^3^
0 restraints	Δρ_min_ = −0.38 e Å^−^^3^

### Special details

**Table:**

Geometry. All e.s.d.'s (except the e.s.d. in the dihedral angle between two l.s. planes) are estimated using the full covariance matrix. The cell e.s.d.'s are taken into account individually in the estimation of e.s.d.'s in distances, angles and torsion angles; correlations between e.s.d.'s in cell parameters are only used when they are defined by crystal symmetry. An approximate (isotropic) treatment of cell e.s.d.'s is used for estimating e.s.d.'s involving l.s. planes.
Refinement. Refinement of *F*^2^ against ALL reflections. The weighted *R*-factor *wR* and goodness of fit *S* are based on *F*^2^, conventional *R*-factors *R* are based on *F*, with *F* set to zero for negative *F*^2^. The threshold expression of *F*^2^ > σ(*F*^2^) is used only for calculating *R*-factors(gt) *etc*. and is not relevant to the choice of reflections for refinement. *R*-factors based on *F*^2^ are statistically about twice as large as those based on *F*, and *R*- factors based on ALL data will be even larger.

### Fractional atomic coordinates and isotropic or equivalent isotropic displacement parameters (Å^2^)

**Table:**

	*x*	*y*	*z*	*U*_iso_*/*U*_eq_	
Cu1	0.72746 (7)	0.75885 (6)	0.84260 (4)	0.03334 (18)	
C1	0.6201 (6)	0.9587 (5)	0.7304 (4)	0.0314 (10)	
C2	0.6438 (5)	0.8236 (4)	0.6230 (3)	0.0285 (9)	
C3	0.6152 (6)	0.7997 (5)	0.4943 (4)	0.0358 (10)	
H3	0.5722	0.8684	0.4626	0.043*	
C4	0.6533 (6)	0.6692 (5)	0.4152 (4)	0.0354 (10)	
H4	0.6343	0.6497	0.3284	0.043*	
C5	0.7184 (5)	0.5677 (5)	0.4608 (3)	0.0340 (10)	
H5	0.7459	0.4815	0.4064	0.041*	
C6	0.7420 (5)	0.5976 (4)	0.5907 (3)	0.0289 (9)	
C7	0.8072 (5)	0.5053 (5)	0.6655 (4)	0.0304 (10)	
C8	0.9050 (6)	0.7252 (5)	1.0648 (4)	0.0397 (11)	
H8	0.9215	0.6365	1.0073	0.048*	
C9	0.9646 (6)	0.7553 (5)	1.1898 (4)	0.0374 (11)	
H9	1.0196	0.6885	1.2168	0.045*	
C10	0.9409 (5)	0.8866 (5)	1.2737 (4)	0.0353 (10)	
H10	0.9783	0.9095	1.3593	0.042*	
C11	0.8617 (5)	0.9847 (4)	1.2313 (3)	0.0275 (9)	
C12	0.8029 (5)	0.9449 (4)	1.1039 (3)	0.0316 (10)	
H12	0.7465	1.0095	1.0748	0.038*	
C13	0.8343 (6)	1.1300 (5)	1.3159 (4)	0.0326 (10)	
N1	0.7036 (4)	0.7233 (4)	0.6650 (3)	0.0286 (8)	
N2	0.8244 (5)	0.8170 (4)	1.0215 (3)	0.0328 (8)	
O1	0.6624 (4)	0.9519 (3)	0.8403 (2)	0.0372 (7)	
O2	0.5679 (4)	1.0631 (3)	0.7065 (2)	0.0413 (8)	
O3	0.8087 (4)	0.5614 (3)	0.7853 (2)	0.0376 (7)	
O4	0.8507 (4)	0.3867 (3)	0.6120 (2)	0.0447 (8)	
O5	0.4365 (4)	0.6488 (3)	0.8620 (2)	0.0461 (8)	
H5B	0.4206	0.6493	0.9369	0.069*	
H5A	0.3623	0.6970	0.8400	0.069*	
O6	0.8709 (5)	1.1433 (3)	1.4365 (2)	0.0436 (8)	
H6	0.8607	1.2267	1.4806	0.065*	
O7	0.7889 (4)	1.2257 (3)	1.2796 (3)	0.0456 (8)	
O1W	0.5969 (5)	1.3367 (3)	0.8942 (3)	0.0705 (11)	
H1WC	0.6522	1.4074	0.8683	0.106*	
H1WD	0.6098	1.2529	0.8447	0.106*	

### Atomic displacement parameters (Å^2^)

**Table:**

	*U*^11^	*U*^22^	*U*^33^	*U*^12^	*U*^13^	*U*^23^
Cu1	0.0503 (3)	0.0311 (3)	0.0194 (3)	0.0134 (3)	0.0010 (2)	0.0071 (2)
C1	0.034 (2)	0.030 (2)	0.031 (2)	0.006 (2)	0.0038 (18)	0.012 (2)
C2	0.032 (2)	0.033 (2)	0.024 (2)	0.010 (2)	0.0030 (17)	0.0127 (19)
C3	0.040 (3)	0.040 (3)	0.032 (2)	0.009 (2)	0.0003 (19)	0.019 (2)
C4	0.043 (3)	0.043 (3)	0.019 (2)	0.005 (2)	0.0024 (18)	0.012 (2)
C5	0.038 (2)	0.034 (3)	0.026 (2)	0.005 (2)	0.0084 (18)	0.006 (2)
C6	0.029 (2)	0.030 (2)	0.025 (2)	0.006 (2)	0.0018 (17)	0.0070 (19)
C7	0.033 (2)	0.029 (2)	0.028 (2)	0.008 (2)	0.0020 (18)	0.007 (2)
C8	0.052 (3)	0.033 (3)	0.034 (2)	0.016 (2)	0.001 (2)	0.006 (2)
C9	0.050 (3)	0.039 (3)	0.027 (2)	0.016 (2)	−0.0017 (19)	0.013 (2)
C10	0.038 (3)	0.044 (3)	0.021 (2)	0.007 (2)	−0.0010 (18)	0.009 (2)
C11	0.031 (2)	0.029 (2)	0.021 (2)	0.0022 (19)	0.0013 (16)	0.0084 (18)
C12	0.041 (3)	0.027 (2)	0.026 (2)	0.009 (2)	0.0022 (18)	0.0079 (19)
C13	0.032 (2)	0.034 (3)	0.026 (2)	0.003 (2)	−0.0011 (18)	0.004 (2)
N1	0.035 (2)	0.030 (2)	0.0204 (17)	0.0055 (17)	0.0008 (14)	0.0087 (15)
N2	0.041 (2)	0.036 (2)	0.0220 (18)	0.0128 (18)	0.0016 (15)	0.0084 (16)
O1	0.058 (2)	0.0322 (17)	0.0246 (15)	0.0160 (15)	0.0012 (13)	0.0098 (13)
O2	0.056 (2)	0.0345 (18)	0.0377 (17)	0.0172 (16)	−0.0006 (14)	0.0149 (14)
O3	0.062 (2)	0.0345 (17)	0.0184 (15)	0.0219 (15)	0.0017 (13)	0.0056 (13)
O4	0.071 (2)	0.0382 (18)	0.0262 (16)	0.0273 (17)	0.0013 (14)	0.0037 (14)
O5	0.051 (2)	0.057 (2)	0.0354 (17)	0.0119 (17)	0.0056 (14)	0.0217 (16)
O6	0.067 (2)	0.0390 (19)	0.0219 (15)	0.0192 (18)	0.0012 (14)	0.0012 (14)
O7	0.066 (2)	0.0359 (19)	0.0338 (17)	0.0190 (17)	−0.0024 (15)	0.0061 (15)
O1W	0.126 (3)	0.044 (2)	0.044 (2)	0.018 (2)	0.033 (2)	0.0157 (17)

### Geometric parameters (Å, °)

**Table:**

Cu1—N1	1.906 (3)	C7—O3	1.284 (4)
Cu1—N2	1.963 (3)	C8—N2	1.335 (5)
Cu1—O1	1.998 (3)	C8—C9	1.370 (5)
Cu1—O3	2.017 (3)	C8—H8	0.9300
Cu1—O5	2.231 (3)	C9—C10	1.370 (5)
C1—O2	1.229 (5)	C9—H9	0.9300
C1—O1	1.277 (4)	C10—C11	1.374 (5)
C1—C2	1.520 (5)	C10—H10	0.9300
C2—N1	1.321 (5)	C11—C12	1.383 (5)
C2—C3	1.387 (5)	C11—C13	1.482 (5)
C3—C4	1.381 (5)	C12—N2	1.333 (4)
C3—H3	0.9300	C12—H12	0.9300
C4—C5	1.371 (5)	C13—O7	1.198 (5)
C4—H4	0.9300	C13—O6	1.327 (4)
C5—C6	1.389 (5)	O5—H5B	0.8500
C5—H5	0.9300	O5—H5A	0.8501
C6—N1	1.329 (4)	O6—H6	0.8200
C6—C7	1.507 (5)	O1W—H1WC	0.8500
C7—O4	1.222 (5)	O1W—H1WD	0.8499
			
N1—Cu1—N2	164.24 (13)	N2—C8—C9	123.2 (4)
N1—Cu1—O1	81.06 (12)	N2—C8—H8	118.4
N2—Cu1—O1	99.27 (12)	C9—C8—H8	118.4
N1—Cu1—O3	80.30 (12)	C10—C9—C8	118.1 (4)
N2—Cu1—O3	97.28 (12)	C10—C9—H9	121.0
O1—Cu1—O3	160.68 (10)	C8—C9—H9	121.0
N1—Cu1—O5	99.42 (11)	C9—C10—C11	120.0 (4)
N2—Cu1—O5	96.26 (12)	C9—C10—H10	120.0
O1—Cu1—O5	94.65 (11)	C11—C10—H10	120.0
O3—Cu1—O5	93.36 (11)	C10—C11—C12	118.4 (4)
O2—C1—O1	126.0 (4)	C10—C11—C13	123.2 (3)
O2—C1—C2	119.6 (3)	C12—C11—C13	118.4 (4)
O1—C1—C2	114.3 (4)	N2—C12—C11	122.1 (4)
N1—C2—C3	120.0 (4)	N2—C12—H12	118.9
N1—C2—C1	111.8 (3)	C11—C12—H12	118.9
C3—C2—C1	128.2 (4)	O7—C13—O6	124.0 (4)
C4—C3—C2	117.1 (4)	O7—C13—C11	124.1 (4)
C4—C3—H3	121.5	O6—C13—C11	111.9 (4)
C2—C3—H3	121.5	C2—N1—C6	123.9 (3)
C5—C4—C3	122.0 (4)	C2—N1—Cu1	117.6 (3)
C5—C4—H4	119.0	C6—N1—Cu1	118.5 (3)
C3—C4—H4	119.0	C12—N2—C8	118.2 (3)
C4—C5—C6	118.0 (4)	C12—N2—Cu1	121.1 (3)
C4—C5—H5	121.0	C8—N2—Cu1	120.6 (3)
C6—C5—H5	121.0	C1—O1—Cu1	114.7 (3)
N1—C6—C5	119.0 (4)	C7—O3—Cu1	115.1 (2)
N1—C6—C7	111.8 (3)	Cu1—O5—H5B	113.4
C5—C6—C7	129.2 (4)	Cu1—O5—H5A	107.7
O4—C7—O3	125.2 (4)	H5B—O5—H5A	108.0
O4—C7—C6	120.6 (3)	C13—O6—H6	109.5
O3—C7—C6	114.3 (3)	H1WC—O1W—H1WD	108.7

### Hydrogen-bond geometry (Å, °)

**Table:**

*D*—H···*A*	*D*—H	H···*A*	*D*···*A*	*D*—H···*A*
O5—H5B···O1W^i^	0.85	1.87	2.715 (4)	175.
O5—H5A···O7^i^	0.85	2.08	2.888 (4)	158.
O6—H6···O4^ii^	0.82	1.79	2.586 (4)	164.
O1W—H1WC···O3^iii^	0.85	2.14	2.983 (4)	172.
O1W—H1WD···O2	0.85	1.95	2.775 (4)	163.

Symmetry codes: (i) −*x*+1, −*y*+2, −*z*+2; (ii) *x*, *y*+1, *z*+1; (iii) *x*, *y*+1, *z*.
